# Appropriate level of alfalfa hay in diets for rearing Simmental crossbred calves in dryland China

**DOI:** 10.5713/ajas.18.0089

**Published:** 2018-05-24

**Authors:** Nobuyuki Kobayashi, Fujiang Hou, Atsushi Tsunekawa, Xianjiang Chen, Tianhai Yan, Toshiyoshi Ichinohe

**Affiliations:** 1 The United Graduate School of Agricultural Sciences, Tottori University, Tottori 680-8550, Japan; 2 Arid Land Research Center, Tottori University, Tottori 680-0001, Japan; 3 State Key Laboratory of Grassland Agro-ecosystems, Key Laboratory of Grassland Livestock Industry Innovation, Ministry of Agriculture, College of Pastoral Agriculture Science and Technology, Lanzhou University, Lanzhou 730000, China; 4 Agri-Food and Biosciences Institute, Belfast BT9 5PX, UK; 5 Faculty of Life and Environmental Science, Shimane University, Matsue 690-8504, Japan

**Keywords:** Alfalfa, Substitution, Feeding Trial, Economic Benefit, Dryland Area in China

## Abstract

**Objective:**

In dryland areas of China, alfalfa hay (AH) is a possible substitute for concentrate feed for beef cattle. To evaluate the potential benefits of this substitution, we studied the effect of the ratio of AH intake to total dry matter (DM) intake on average daily body-weight gain (ADG), dietary energy utilization status, and economic benefit in Gansu province.

**Methods:**

In each of two feeding trials in 2016 (trial 1 [T1], July 3 to 17; trial 2 [T2], August 15 to September 23), crossbred male Simmental calves were allocated to low AH (LA), medium AH (MA), and high AH (HA) feeding groups (n = 4 per group). The target ADG was set as 1 kg for both trials. In a one-way-layout design based on conventional feeding practices in the province, calves received diets containing the different AH amounts, with a constant ratio of corn stover:total DM and decreasing rations of concentrate feed proportional to the increase in AH. Calves in T1 received AH at 15% (T1-LA), 23% (T1-MA), or 31% (T1-HA) of their dietary DM allowances; those in T2 received 9% (T2-LA), 24% (T2-MA), or 34% (T2-HA) AH.

**Results:**

Among the T1 groups, both ADG and economic benefit were highest in T1-LA; whereas in T2, they were higher in the T2-LA and T2-MA groups than in T2-HA. Energy digestibility did not significantly differ among the groups in either trial. The dietary AH inclusion ratios of 14% in the warm season and 8% to 21% in the cool season appeared to yield optimal ADG, metabolizable energy intake, and economic benefit.

**Conclusion:**

Low-level inclusion of AH, ranging from 8% to 21%, is a practical approach for beef cattle feeding. This modified feeding regimen likely will promote increased growth performance during the fattening stage of beef steers in dryland areas of Gansu province, China.

## INTRODUCTION

The amounts of concentrate feed ingredients and forages imported into China have increased due to the increased demand for animal feed. Because livestock grazing has been prohibited to prevent the desertification of natural pastures in many provinces of China [1], feeding systems integrating locally produced roughage that provide a high level of animal production performance are required for confined beef cattle. As a roughage source in the drylands of China, alfalfa is recommended because of its drought tolerance, nutritive value [2,3], feasible economic return to beef farmers, and nationwide cultivation [4].

Simmental crossbreds are a common type of beef cattle, particularly in Gansu Province, which is a major beef production area in the drylands of China [5]. The dietary inclusion of alfalfa hay (AH) at low levels (*i.e*., 8.1% and 22.6% of total dry matter intake [DMI]) for growing Simmental crossbred calves in Gansu Province did not reduce energy digestibility and metabolizability and had no detrimental effects on economic benefit and CH_4_ production, as compared with a conventional concentrate-based diet [4]. Substitution of alfalfa, a high-quality domestic roughage, for concentrate feed (C) at an appropriate proportion in the ration would increase animal feed self-sufficiency in China and yield economic benefits and improve production performance for farmers.

A previous, preliminary study [4] assessed the effects of dietary AH level (including no AH) on the performance and economic benefit of beef cattle. However a broad range of AH inclusion levels must be evaluated to optimize the ration formulation for use in the dryland region of Gansu Province: we performed two feeding trials to study the effect of the ratio of AH intake to total DMI on the average daily body-weight gain (ADG), dietary energy utilization status, and economic profitability of crossbred Simmental cattle in Gansu Province. We then used our current data and those of the previous report [4] to discuss the optimal ratio of AH to total DM allowance for substituting AH for C in this context.

## MATERIALS AND METHODS

### Animal care

The crossbred Simmental calves used for this study were cared for according to the provisions of the guide for care and use of laboratory animals [6] and under the supervision of Gansu Province Animal Care Committee, throughout the experimental periods.

### Study site

The trials were conducted at Linze Research Station (College of Pastoral Agriculture Science and Technology, Lanzhou University, China), which is located in Linze County of Gansu Province. The research station is situated at 39.24°N, 100.06°E and at an elevation of 1,390 m above sea level. The annual mean precipitation is about 130 mm in this region, and the total precipitation was 50 mm in 2016, all of which occurred during March through October. The annual average temperature during that year was 6.3°C (data supplied by the Linze Research Station). The study site is categorized as a typical arid zone.

### Cattle, applied diets, and feeding management

Two feeding trials were conducted in 2016 (T1 [July 3 to 17] was during the warm season; T2 [August 15 to September 23] was during the cool season), with the aim of achieving 1-kg ADG. Prior to the trials, cattle were adapted to the experimental feeding regimens (T1 [June 21 to July 2]; T2 [July 26 to August 14]). Male crossbred Simmental calves (n = 12 per trial) were purchased from a local market for T1 (age, 6 months; body weight [BW], 126.2±8.0 kg) and T2 (age, 7 months; BW, 159.4±9.9 kg). The average air temperatures during T1 and T2 were 19.8°C and 16.1°C, respectively. When designing the experimental feeding regimens, we considered the conventional feeding practices of beef farmers in Gansu Province, *i.e.*, higher amounts of C in the cool season than the warm season. The 12 calves in each trial were assigned to one of three groups (low AH [LA], medium AH [MA], and high AH [HA]; n = 4 per group) so that initial BW did not differ significantly among groups. These three groups differed in the amount of AH fed. All calves were fed forage diets comprising corn stover (CS) and AH, supplemented with C. The diets of the T1-LA and T2-MA groups were designed based on the low-level AH mixtures used for the feeding trials conducted in 2015 [4]; these dietary allowances (T1-LA and T2-MA) were regarded as being practically appropriate for the drylands of Gansu Province, China. In T1, to assess the effect of adding more of the AH mixture than the proportion used in the low-AH mixture group in that report [4], the calves in the MA group (T1-MA) received an increased amount of AH with CS and C, whereas those in the HA group (T1-HA) received a proportionately greater amount of AH with CS and C. In T2, to assess the effect of changing the proportion of the AH mixture from the quantity in the low-level AH feeding [4], the calves received CS, C, and a decreased amount of AH in the LA group but an increased amount of AH in the HA group.

The experimental diet for each group was designed to provide sufficient metabolizable energy (ME) and metabolizable protein (MP) for 1-kg ADG for a bull calf according to a published estimation equation and values [7,8] and the calves’ BW (measured weekly). Reported tabular values [9] were used for converting the reported DE values of CS, AH, and the feed ingredients of C [7] into ME concentrations. In addition, confirmed nutritional values of the commercial concentrate components of C were used to calculate the ME concentration of C. Because the ME requirement for 1-kg ADG per kg^0.75^ BW of calf was greater for T2 than that for T1 [7], the experimental diets in T2 with relatively greater amount of C were designed to meet the ME requirement within the calculated DMI [8]. In a one-way-layout design, calves were fed the diets comprising AH (as a percentage of DM) at 15% (T1-LA), 23% (T1-MA), 31% (T1-HA), 9% (T2-LA), 24% (T2-MA), or 34% (T2-HA); a constant ratio (as a percentage of DM) of CS; and decreasing quantities of C in proportion to the increase in AH. The CS and AH used in both trials were cultivated at Linze Research Station. The CS used in both trials was harvested in September 2015. The AH used in both trials was harvested in July 2015 (second-cutting hay) and September 2015 (third-cutting hay). The C comprised commercial concentrate (30%), wheat bran (10%), and corn grain (60%). The commercial concentrate was composed of soybean meal, sunflower meal, rape seed meal, cotton seed meal, urea, sodium chloride, and a vitamin and mineral premix (precise composition unavailable). The corn grain was produced at Linze Research Station in September 2015. Throughout the feeding trials, the calves were housed individually and had free access to fresh water and mineral blocks. They were fed a mix of coarsely chopped CS and AH (5 to 10 cm in length) twice daily (07:30 and 19:30) and a separate meal of C once daily (at 14:30) in separate troughs for each animal.

### Measurements and sample collection

The calves were weighed weekly during each trial. The amount of feed provided to each calf was calculated weekly according to each animal’s updated BW. Throughout both trials, the amounts of feed offered to the calves and the refusals were weighed and recorded daily to calculate the daily feed intake. Immediately before and after each trial, jugular blood samples (10 mL per calf) were collected after the morning feeding of roughage. Immediately after collection, the samples were centrifuged at 600×g for 10 min at room temperature, and the plasma was stored at −20°C. In addition, representative samples of CS, AH, and C were collected several times during each feeding trial for chemical composition analysis of the feed.

After completion of T1, all 12 calves were evaluated in ventilated open-circuit respiration chambers. During the 5-day acclimation period, representative fecal samples were collected every morning for 3 days to determine the daily fecal gross energy (GE) excretion of calves in each treatment group. Daily fecal excretion was estimated by using acid detergent lignin (ADL) as an internal marker, to determine digestibility. ME intake (MEI) was calculated by using the conversion ratio of 0.88 for DE into ME obtained during the trials in 2015 [4]. After the acclimation period, O_2_ consumption and CO_2_ and CH_4_ production were measured for 48 h (two consecutive 24-h measurements for each calf) by using a paramagnetic-based O_2_ gas analyzer and an infrared absorption-based gas analyzer (CO_2_ and CH_4_) (VA-3000, Horiba, Kyoto, Japan). The average BW of the calves at the start of the respiratory measurements was 144.4±14.2 kg. During the respiration trials, calves received the same diet and on the same schedule as during the feeding trials. The daily heat production (HP) of each animal was calculated by using a reported equation [10]. The ME for maintenance (MEm) of male calves was estimated by using a linear regression equation between the net energy (NE) intake (obtained by subtracting HP from MEI) and MEI, with both expressed on the basis of metabolic body size (kg^0.75^ BW), as reported [11]. At the end of T2, the representative fecal samples were likewise collected for estimating fecal GE excretion of calves, but the respiratory measurements were not performed due to technical problems with the measuring apparatus.

### Chemical analysis

Collected feed and fecal samples were dried in a forced-air oven at 60°C, ground, and sieved to pass through a 1-mm screen. Concentrations of DM, crude protein (CP), ash-free neutral detergent fiber (NDFom), and ADL in the dried samples were determined by using standard methods [12,13]. Concentrations of GE were determined by using a bomb calorimeter (CA-4AJ, Shimadzu, Kyoto, Japan). Plasma concentrations of the following metabolites were measured by using commercial kits: glucose (Glucose C-test, Wako Pure Chemical Industries, Osaka, Japan), non-esterified fatty acid (NEFA) (NEFA C-test, Wako Pure Chemical Industries, Japan), blood urea nitrogen (BUN) (C013-2, Nanjing Jiancheng Bioengineering Institute, Nanjing, China), and β-hydroxybutyrate (BHBA) (3-HB auto, Wako Pure Chemical Industries, Japan). A spectrophotometer (Cary 60, Agilent Technologies, Santa Clara, CA, USA) was used for performing the analyses.

### Economic analysis

To examine the economic feasibility of substituting AH for C, differences in the feeding costs for each of the dietary treatment groups in T1 and T2 were calculated. Feed costs were calculated by using the sum of the expenses incurred for the purchase of AH and commercial concentrate at their market prices (1.70 yuan/kg for AH and 2.54 yuan/kg for C) and the daily intake of AH and C for each calf in T1 and T2. The CS feed cost was not considered, because CS typically is prepared by each farmer as a forage source for feeding his cattle. We regarded the other costs associated with feeding as comparable between all the groups in both trials. In addition, the economic benefit of the calves’ ADG was estimated by subtracting the feed cost from the expected income (profit) from the ADG values calculated according to the market price of the calves (22 yuan/kg BW). The estimates then were converted at the rate of US$1 = 6.68 yuan (based on the average of values for the periods of July 3 to 17 and of August 15 to September 23, 2016).

### Statistical analysis

Differences in means among the three groups in each trial or between the groups in the current study and those previously reported [4] were evaluated by using one-way analysis of variance and Tukey’s test after the normality and homoscedasticity of data distribution were evaluated (Kolmogorov–Smirnov and Barlett tests, respectively). Possible seasonal differences in the efficiency of energy and N utilization were not considered because of differences in the feeding regimens applied in T1 and T2. All statistical analyses were performed by using R statistical software (version 3.1.1, the R Foundation for Statistical Computing, Vienna, Austria). Significance was declared at p≤0.05, and trends were identified at 0.05<p≤0.10.

## RESULTS AND DISCUSSION

### Chemical composition of diets

The GE concentration and chemical composition of feed ingredients are shown in Table 1. The CP concentration of C was higher in T2 than in T1, because the commercial concentrate used in T1 was unavailable for T2, necessitating replacement with a different product. The NDFom concentrations of AH (46.9% for T1 and 52.4% for T2, as a percentage of DM) were higher than some values reported previously (36.0% to 39.3%) [9] but were similar to others (52.2% to 52.8%) [4].

Daily nutrient allowances and estimated chemical compositions of the experimental diets of the calves at the start of each trial are shown in Table 2. In both trials, the estimated dietary NDFom concentration tended to increase as the proportion of AH fed increased because of higher concentrations of NDFom in AH than in C (Table 1). The ADL concentrations in all of the diets in the current study exceeded the reported value of 2% (as a percentage of DM) [14] and were sufficiently high for their use as an internal marker to estimate fecal DM excretion. The concentrations of CP in CS (5.1% and 4.4% DM for T1 and T2, respectively) (Table 1) and those of NDFom (72.6% and 77.6% DM for T1 and T2, respectively) were consistent with the report that crossbred Simmental male calves require supplementation with a concentrate or leguminous forage when they are fed CS with low CP concentration as the basal forage [4].

### Feed intake, feed and energy utilization efficiency, and growth performance

Feed and nutrient intake and digestibility for each group are shown in Table 3. DMI (in kg/d and % BW) did not differ between groups in either T1 or T2 and was consistent with a study reporting that dietary substitution with AH did not reduce total DMI [4]. In T1, the C intake did not decrease from T1-LA to T1-HA in proportion to the increase in the AH intake. Consequently, the ratio of C intake (on a DM basis) to total DMI did not gradually decrease as AH intake increased, and the ratio of roughage (CS and AH) intake to total DMI did not differ among the three groups in T1 (p = 0.51). We attributed the lack of proportional decrease in the C intakes in T1-MA and T1-HA to the calves’ preference for C rather than AH, which was facilitated by separately feeding C and AH at different times. The ratio of roughage intake to total DM allowance was reportedly 12% when rice straw (as roughage) and concentrate (ingredients not specified) were fed separately and *ad libitum* to fattening steers [15]. In our study, the designed ratios of roughage (CS and AH) to total DM allowance in T1 (44% to 58%, Table 2) were much greater and might have caused the preferred intake of C by calves. By contrast in T2, according to the increase in the ratios of AH intake to total DMI (p<0.05) (Table 3), the C intakes tended to decrease proportionally (p<0.10). The digestion coefficients of DM, CP, and NDFom did not differ among the three groups in either T1 or T2. Decreased NDFom digestibility with concurrent increased NDFom intake is the suspected source of an AH-associated decrease in energy retention for ADG [16]. The increase in NDFom intake from T2-LA to T2-MA might have induced the observed decline in ADG (Table 4). In both trials, energy digestibility did not differ among the three groups (p>0.10) (Table 3), and DE intake showed no clear trend as the ratio of AH intake to total DMI increased.

The growth performance and economic benefit of each group are shown in Table 4. The ADG in the T1-LA and T1-HA groups tended to be higher than in the T1-MA group (p< 0.10). The ADG in T1-MA did not meet the target, which was achieved in the other groups in T1. In T2, the ADG was numerically greater in the T2-LA and T2-MA groups than in the T2-HA group (p = 0.12). In both trials, the LA groups, which received AH at low levels (*i.e.*, 14.2% and 7.8% of total DMI), achieved the desired ADG (1 kg/d) and the highest ADG in each trial.

**Figure 1:**
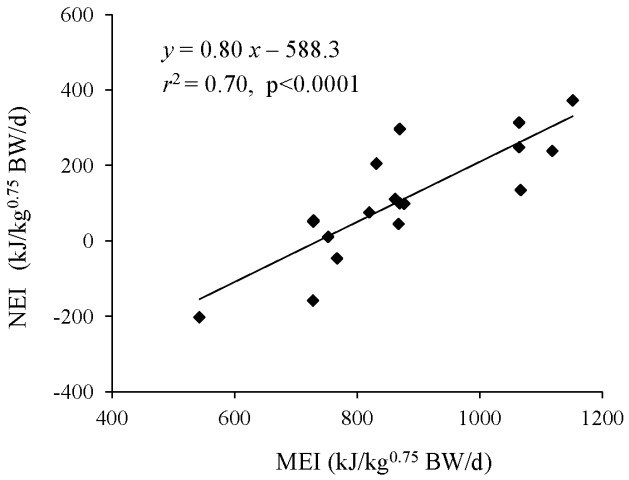
Linear regression of net energy intake (NEI, *y*) and metabolizable energy intake (MEI, *x*) of calves (mean BW, 148.4 kg) in Trial 1. NEI was estimated as MEI – heat production (HP). Metabolizable energy for maintenance was the interpolant of *x* at the point where *y* is 0.

The MEm of 737 kJ/kg^0.75^ BW/d calculated in T1 (Figure 1) was higher than the values previously reported for male Simmental calves [4]. To ensure their energy requirements, crossbred Simmental bull calves reportedly require greater energy allowances than those of other breeds [4]; our current data are consistent with this previous finding. However, the ratio of ADG to MEI (ADG/ME, g/MJ/d) in the LA groups (34.1 in T1-LA and 27.2 in T2-LA) exceeded or approximated that reported for Xiangzhong Black bulls (31.1 g/MJ/d) [17]. The ME utilization efficiency for ADG in appropriately fed crossbred Simmental male calves yields similar growth performance to that of indigenous Xiangzhong Black cattle. That is, efficient utilization of ME for BW gain resulted in the high ADG/ME.

### Economic evaluation of feeding AH to calves

In T1, economic benefit was numerically highest in the LA group (Table 4). Economic benefit declined slightly from T1-LA to T1-MA, reflecting an increase in feed cost (for T1-LA vs T1-MA) due to the increased AH and C intakes. In T2, economic benefit was numerically highest in the LA group because the significant increase in AH intake (p<0.05) was accompanied by a relatively small decrease in C intake (p<0.10) from T2-LA to T2-HA (Table 3). Both LA groups achieved the 1-kg ADG target by consuming a diet that included a low level of AH (*i.e.*, 7.8% to 14.2% on a DM basis) added to C-based ration. As reported previously [4], incorporating small proportions of AH into a concentrate-based regimen (T1-LA and T2-LA in the current study) appears to be acceptable in terms of economic feasibility for feeding Simmental beef calves.

### Blood metabolites

The post-trial values of blood metabolites for each group are shown in Table 5. Blood glucose, NEFA, and BHBA concentrations in all groups of both trials were within physiologically normal ranges (2.50 to 3.89 mmol/L, 200 to 800 μEq/L, and less than 1,200 μmol/L, respectively) [18,19] and indicated sufficient energy supply.

### Appropriate ratio of AH to total DM allowance

We analyzed our current data regarding the optimal ratio of AH intake (on a DM basis) to total DMI in the context of previous results of feeding trials conducted during 2015 at Linze Research Station [4]. Because of the differences between the feeding regimens for the 2 trials in both years, we compared T1 (in the current study) with the trial performed during the 2015 warm season [4] and compared T2 with the trial completed during the 2015 cool season [4].

In the T1 groups (Table 6), the ADG at the AH-intake ratio of 19.1% was lower than the ADG in the other groups (p< 0.05). The ADG gradually increased when the AH-intake ratio was ≤14.2%. The trend in values of feed conversion ratio (FCR) appeared to be opposite to that obtained for the ADG values. The FCR was lower for the AH-intake ratio of 14.2% and 24.2%, supporting the difference from that for a ratio of 19.1% (p<0.05). At the AH-intake ratio of 19.1%, the roughage to total DMI ratio (61.4%) was significantly higher than in the other five T1 groups (33.4% to 47.8%) (p<0.01). The roughage: intake ratio affects the ADG and FCR [20–22]; the roughage: intake ratio at the AH-intake ratio of 19.1% in the current study was much higher than that reported as appropriate for Holstein steers fed commercial concentrate and rice straw (12%) [15] and that for Japanese Black steers fed commercial concentrate, timothy grass (*Phleum pretense*), and corn silage (50%) [23]. The increase in FCR from the AH-intake ratio of 14.2% (roughage:intake ratio, 45.3%) to the AH-intake ratio of 19.1% (roughage:intake ratio, 61.4%) indicates that excessive roughage intake increased the FCR and reduced the ADG. The ADG and FCR did not differ between the groups with AH-intake ratios of 24.2% and 14.2% (p = 0.99 for both ADG and FCR), likely because the roughage:intake ratio for the former (47.8%) did not markedly differ from that for the latter (45.3%). When AH is substituted for C in diets with a constant ratio of CS to total DMI, adding more AH than that used for the group with the AH-intake ratio of 14.2% risks reducing ADG. The FCR of feedlot cattle is typically less than 6 [24], and FCR values of 8.3 in Chongqing (Western China) and of 6.4 to 7.1 in Inner Mongolia (feeding style not specified) are reported [25]. Even though FCR values are typically lower for younger growing calves than for older animals [8,21], the FCR value (3.16) achieved by using an AH-intake ratio of 14.2% indicates a particularly high level of feeding efficiency in Simmental crossbred beef cattle. The inclusion of AH at ≤14.2% and 14.2% of total DMI seemed appropriate in terms of ADG and FCR, respectively. The MEI did not differ between groups at AH-intake ratios of ≤14.2%. The concentrations of blood metabolites indicate sufficient energy supply at the AH-intake ratio of 14.2% (Table 5). Previously, reduction in MEI due to the mixture of AH into C-based diets decreased ADG or energy utilization efficiency when the ratio of AH incorporated was 19.1% [4]. The energy supply in the groups with the AH-intake ratio of ≤14.2% may have met the NE requirement for both maintenance and 1-kg ADG for growing Simmental male calves. Economic benefit was higher when the AH-intake ratio was 8.1% or 14.2% than with a ratio of 19.1% (p< 0.05); this increase relative to the increased amount of AH (to a maximum of 14.2%) was similar to that of ADG. We did not recommend a specific range of AH-intake ratio effective for optimizing the digestion coefficients of DM, CP, and NDFom because of the lack of significant difference in these digestion coefficients among all T1 groups (p = 0.68 to 0.88).

In the T2 groups (Table 7), ADG was numerically the highest at the AH-intake ratio of 7.8%. The ADG decreased and the FCR increased as the AH-intake ratio increased above 7.8%. The FCR values (3.55 to 3.71) associated with the AH-intake ratios of 7.8% and 21.1% indicate high feeding efficiency for Simmental crossbred beef cattle in T2, as seen in T1. The MEI at the AH-intake ratio of 7.8% and 21.1% was significantly higher than that at the ratio of 0% (p<0.05) and slightly higher than that at the ratio of 22.6% (p = 0.17). The groups with the ratios of 7.8% and 21.1% achieved the target 1-kg ADG and seemed to provide the calves’ energy requirements for maintenance and 1-kg ADG. The AH inclusion at 7.8% to 21.1% of total DMI was therefore appropriate in terms of energy intake and efficiency. Economic benefit was higher at the AH-intake ratio of 7.8% than at ratios of 0% or 38.1% (p<0.05) and did not differ among the groups with ratios of 7.8% to 30.1%. This range included the ratio of 14.2%, which was associated with the highest economic benefit in the T1 groups. In the T2 groups, the digestion coefficients of DM, CP, and GE decreased slightly at AH-intake ratios of ≥7.8%, consistent with findings of previous studies [26,27], but the decrease was not significant (p>0.10) and did not appear to affect growth performance (*i.e.*, ADG and FCR).

Data from the current study and the previous report [4] thus support the following ratios of AH intake to total DMI as appropriate for male Simmental beef cattle according to the following criteria for T1 and T2 groups (T2 groups received more C than T1 groups), respectively: ADG, ≤14.2% and ≥7.8%; FCR, 14.2% and 7.8% to 30.1%; energy intake and utilization efficiency, ≤14.2% and 7.8% to 21.1%; and economic benefit, 8.1% to 14.2% and 7.8% to 30.1%. The ratios of 14.2% and 7.8% to 21.1% are appropriate for the warm and cool seasons, respectively. We therefore recommend low-level inclusion of AH (*i.e.*, 8% to 21% of total DM) as a practical feeding method that can achieve greater than 1-kg ADG in growing beef cattle and that likely will promote subsequent robust growth performance during the fattening stage, in dryland areas of Gansu province, China. However, more studies are required to test current findings through further *in vivo* trials.

## Figures and Tables

**Table 1:** Gross-energy concentrations and chemical compositions of feed ingredients in experimental diets for Simmental beef calves in Gansu province, China

Items	GE (kJ/g DM)	Chemical composition (% DM)				

		OM	CP	ADFom	NDFom	ADL
Trial 11)						
Corn stover	16.2	89.6	5.1	42.8	72.6	5.3
Alfalfa hay2)	17.4	91.8	13.5	34.9	46.9	7.6
Concentrate3)	18.1	97.1	12.7	4.5	15.0	1.0
Trial 21)						
Corn stover	16.4	92.3	4.4	43.7	77.6	5.2
Alfalfa hay2)	17.1	90.4	12.3	38.7	52.4	8.3
Concentrate3)	17.5	93.6	18.0	6.8	18.1	1.4

GE, gross energy; DM, dry matter; OM, organic matter; CP, crude protein; ADFom, ash-free acid detergent fiber; NDFom, ash-free neutral detergent fiber; ADL, acid detergent lignin.

1) Trial 1 was conducted from July 3 to 17, 2016 (samples were collected on July 3); Trial 2 ran from August 15 to September 23, 2016 (samples were collected on August 15).

2) A 50:50 mixture of alfalfa hay harvested in July and September 2015 was used as feed and analyzed.

3) The feed concentrate comprised 60% corn grain, 30% commercial concentrate, and 10% wheat bran.

**Table 2:** Feed allowances and chemical composition of experimental diets formulated for Simmental beef calves

Items	Trial 11)			Trial 21)		
	
	T1-LA	T1-MA	T1-HA	T2-LA	T2-MA	T2-HA
Feed allowance2) (kg DM/d)						
Corn stover	1.2	1.2	1.2	1.3	1.3	1.3
Alfalfa hay	0.6	1.0	1.4	0.4	1.2	1.8
Concentrate	2.3	2.1	1.9	3.0	2.5	2.2
Estimated chemical composition3) (% DM)						
CP	10.6	10.8	10.9	13.8	13.1	12.7
NDFom	36.6	38.4	40.4	37.1	42.1	44.4
ADL	3.2	3.7	4.2	3.0	4.1	4.7

T1, trial 1; T2, trial 2; DM, dry matter; CP, crude protein; NDFom, ash-free neutral detergent fiber; ADL, acid detergent lignin.

1) T1-LA and T2-LA, low level of alfalfa hay feeding; T1-MA and T2-MA, medium level of alfalfa hay feeding; T1-HA and T2-HA, high level of alfalfa hay feeding.

2) Calculated by using a published equation (AFRC [8]) based on the initial average BW of male calves in Trial 1 (126.2 kg) and Trial 2 (159.4 kg) to meet the metabolizable energy requirement for an average daily body-weight gain of 1 kg.

3) Values were estimated according to the chemical composition of feed ingredients (Table 1) and the composition of ingredients in the experimental diets.

**Table 3:** Feed and nutrient intake, digestibility, and energy utilization in Simmental crossbred beef calves with different levels of alfalfa hay in their diets

Items	Trial 11)					Trial 21)				
	
	T1-LA	T1-MA	T1-HA	SEM	p value	T2-LA	T2-MA	T2-HA	SEM	p value
Feed intake										
Corn stover (kg DM/d)	1.0^d^	0.9d,e	0.7^e^	0.08	0.06	1.3^d^	1.3^d^	1.2^e^	0.03	0.01
Alfalfa hay (g DM/d)	0.5^b^	0.7a,b	0.8^a^	0.06	0.02	0.4^c^	1.1^b^	1.6^a^	0.04	0.0005
Concentrate (kg DM/d)	1.9	2.0	1.7	0.14	0.34	3.4^d^	2.8^e^	2.5^f^	0.08	0.0005
Total DMI (% BW)	2.63	2.57	2.47	0.17	0.58	2.73	2.73	2.84	0.04	0.09
Nutrient intake (kg DM/d)										
CP	0.36	0.39	0.37	0.03	0.63	0.71	0.69	0.69	0.02	0.66
NDFom	1.28	1.26	1.18	0.09	0.74	1.85^b^	2.09^a^	2.18^a^	0.05	0.004
Digestibility (%)										
DM	58.7	63.2	66.2	4.35	0.45	69.6	64.0	66.1	2.37	0.31
CP	49.5	51.2	60.5	5.72	0.39	71.6	66.8	68.1	2.89	0.51
NDFom	39.6	50.3	48.0	3.95	0.19	58.2	51.1	57.6	2.62	0.16
Energy utilization										
GE intake (kJ/kg^0.75^ BW/d)	1,555.1	1,547.8	1,470.5	70.1	0.65	1,736.1	1,739.1	1,793.0	21.9	0.17
DE intake (kJ/kg^0.75^ BW/d)	946.6	1,000.8	992.2	84.8	0.89	1,226.8	1,135.9	1,208.6	45.0	0.30
Energy digestibility (DE/GE, %)	61.0	64.2	67.5	4.11	0.56	70.6	65.4	67.4	2.43	0.35

T1, trial 1; T2, trial 2; SEM, standard error of the mean; DM, dry matter; DMI, dry matter intake; BW, body weight; CP, crude protein; NDFom, ash-free neutral detergent fiber; GE, gross energy; DE, digestible energy.

1) T1-LA and T2-LA, low level of alfalfa hay feeding; T1-MA and T2-MA, medium level of alfalfa hay feeding; T1-HA and T2-HA, high level of alfalfa hay feeding.

a–f Means with different superscripts within each trial and row differ significantly (a–c p≤0.05) or tend to differ (d–f 0.5<p≤0.10).

**Table 4:** Growth performance and economic benefit of Simmental beef calves with different levels of alfalfa hay in their diets

Items	Trial 11)					Trial 21)				
	
	T1-LA	T1-MA	T1-HA	SEM	p value	T2-LA	T2-MA	T2-HA	SEM	p value
Growth performance										
ADG (kg/d)	1.09	0.92	1.06	0.07	0.27	1.46	1.40	1.23	0.10	0.30
Feed conversion ratio, (kg DMI/kg ADG)	3.16a,b	3.98^a^	3.12^b^	0.27	0.09	3.55	3.71	4.31	0.31	0.29
Economic benefit2) (US$/d/head)	2.65	1.97	2.54	0.23	0.13	3.28	3.14	2.58	0.34	0.35

T1, trial 1; T2, trial 2; SEM, standard error of the mean; ADG, average daily body-weight gain; DMI, dry matter intake.

1) T1-LA and T2-LA, low level of alfalfa hay feeding; T1-MA and T2-MA, medium level of alfalfa hay feeding; T1-HA and T2-HA, high level of alfalfa hay feeding.

2) Calculated based on the results for feed intakes (Table 3) obtained in the feeding trials.

a,b Means with different superscripts within each trial and row tend to differ (0.5<p≤0.10).

**Table 5:** Post-trial blood metabolites in Simmental beef calves with different levels of alfalfa hay in their diets

Items	Trial 11)				Trial 21)			
	
	T1-LA	T1-MA	T1-HA	SEM	T2-LA	T2-MA	T2-HA	SEM
Glucose (mmol/L)	3.44	3.15	2.61	0.27	4.47	4.37	4.53	0.10
NEFA (μEq/L)	497.28	426.93	473.02	85.88	214.33	221.24	211.30	7.91
BHBA (μmol/L)	233.58	81.44	116.15	54.81	195.81^b^	261.67a,b	367.08^a^	47.41

T1, trial 1; T2, trial 2; SEM, standard error of the mean; NEFA, non-esterified fatty acid; BHBA, β-hydroxybutyrate.

1) T1-LA and T2-LA, low level of alfalfa hay feeding; T1-MA and T2-MA, medium level of alfalfa hay feeding; T1-HA and T2-HA, high level of alfalfa hay feeding.

a,b Means with different superscripts within each trial and row tend to differ (0.5<p≤0.10).

**Table 6:** Growth performance, energy intake, and economic benefit of Simmental crossbred male calves with different alfalfa-hay intakes (August to September 2015 and July 2016)

Items	Alfalfa-hay intake (on a DM basis)/total DMI (%)						SEM	p value

	0	8.1	14.2	19.1	19.4	24.2		
ADG (kg/d)	0.94a,b	1.03^a^	1.09^a^	0.63^b^	0.92a,b	1.06^a^	0.09	0.0005
Feed conversion ratio (kg DMI/kg ADG)	5.01a,b	4.89b,c	3.16^d^	8.42^a^	3.98b,c,d	3.12^d^	0.84	0.0005
MEI (kJ/kg0.75 BW/d)	895.2	875.1	831.01)	795.3	878.61)	871.01)	87.9	0.96
Economic benefit (US$/d/head)	1.60a,b	2.03^a^	2.65^a^	0.91^b^	1.97a,b	2.54^a^	0.28	0.0005

DMI, dry matter intake; SEM, standard error of the mean; ADG, average daily body-weight gain; DM, dry matter; MEI, metabolizable energy intake.

Values of ADG, feed conversion ratio, MEI, and economic benefit at the alfalfa-hay intake/total DMI of 0%, 8.1%, and 19.1% are those reported in [4], whereas those for 14.2%, 19.4%, and 24.2% were obtained in the current study.

1) Calculated by using the ratio for converting DE into ME reported in [4].

a–d Means with different superscripts within each row differ significantly (p≤0.05).

**Table 7:** Growth performance, energy intake, and economic benefit of Simmental crossbred male calves with different alfalfa-hay intakes (September to October 2015 and August to September 2016)

Items	Alfalfa-hay intake (on a DM basis)/total DMI (%)						SEM	p value

	0	7.8	21.1	22.6	30.1	38.1		
ADG (kg/d)	0.69^b^	1.46^a^	1.40^a^	1.20^a^	1.23^a^	1.15^a^	0.13	0.002
Feed conversion ratio (kg DMI/kg ADG)	5.83^a^	3.55^b^	3.71a,b	4.58a,b	4.31a,b	6.09^a^	0.66	0.02
MEI (kJ/kg^0.75^ BW/d)	744.3^c^	1,077.0^a^,1)	997.2a,b, 1)	857.3b,c	1,061.0^a^, 1)	986.8a,b	39.5	0.0005
Economic benefit (US$/d/head)	0.68^c^	3.28^a^	3.14a,b	1.96a,b,c	2.58a,b	1.50b,c	0.45	0.001

ADG, average daily body-weight gain; DM, dry matter; DMI, dry matter intake; MEI, metabolizable energy intake, SEM, standard error of the mean.

Values of ADG, feed conversion ratio, MEI, and economic benefit at the alfalfa-hay intake/total DMI of 0%, 22.6%, and 38.1% are those reported in [4], whereas those for 7.8%, 21.1%, and 30.1% were obtained in the current study.

1) Calculated by using the ratio for converting DE into ME reported in [4].

a–d Means with different superscripts within each row differ significantly (p≤0.05).
